# From Abnormal Hippocampal Synaptic Plasticity in Down Syndrome Mouse Models to Cognitive Disability in Down Syndrome

**DOI:** 10.1155/2012/101542

**Published:** 2012-07-12

**Authors:** Nathan Cramer, Zygmunt Galdzicki

**Affiliations:** Department of Anatomy, Physiology, and Genetics, F. Edward Hébert School of Medicine, Uniformed Services University of the Health Sciences, Bethesda, MD 20814, USA

## Abstract

Down syndrome (DS) is caused by the overexpression of genes on triplicated regions of human chromosome 21 (Hsa21). While the resulting physiological and behavioral phenotypes vary in their penetrance and severity, all individuals with DS have variable but significant levels of cognitive disability. At the core of cognitive processes is the phenomenon of synaptic plasticity, a functional change in the strength at points of communication between neurons. A wide variety of evidence from studies on DS individuals and mouse models of DS indicates that synaptic plasticity is adversely affected in human trisomy 21 and mouse segmental trisomy 16, respectively, an outcome that almost certainly extensively contributes to the cognitive impairments associated with DS. In this review, we will highlight some of the neurophysiological changes that we believe reduce the ability of trisomic neurons to undergo neuroplasticity-related adaptations. We will focus primarily on hippocampal networks which appear to be particularly impacted in DS and where consequently the majority of cellular and neuronal network research has been performed using DS animal models, in particular the Ts65Dn mouse. Finally, we will postulate on how altered plasticity may contribute to the DS cognitive disability.

## 1. Introduction

Down syndrome (DS) results from the triplication of genes on human chromosome 21 (Hsa21) and is associated with a range of phenotypes including craniofacial changes [1, 2], cardiac defects [3], susceptibility to leukemia but with reduced occurrence of solid cancers [4, 5], and intellectual disability [6, 7]. While the presence and severity of these individual phenotypes vary among DS individuals, every individual with DS has some degree of cognitive impairment. These impairments limit the independence of DS subjects and adversely impact their quality of life. Consequently, understanding the genetic causes of cognitive dysfunction in DS has been the focus of much research in this field.

The phenomenon of synaptic plasticity has been strongly linked to cognitive processes, such as learning and memory [8, 9]. Synaptic plasticity refers to the dynamic nature of synapses, sites of communication between neurons, in which the structure, composition, or function of the synapse changes in response to network activity. Depending on the timing and strength of pre- and postsynaptic activity, synapses can either be strengthened or weakened providing a potential mechanism for memory formation and storage [10]. Structurally, synaptic connections on excitatory neurons are typically formed on the heads of dendritic spines [11]. The morphology of the spines enables compartmentalization of signaling cascades and facilitates manipulation of the structure and composition of the cell membrane by second messenger systems [12, 13]. Thus, not only is the number of spines important, as individual locations for excitatory synaptic transmission, but the shape of the individual spines also has a critical functional role.

The link between synaptic plasticity and cognitive processes such as learning and memory is frequently studied within the hippocampus, a structure involved in diverse cognitive processes such as those related to acquisition, coding, storing, and recalling information in physical or perceived spatial environments [14–16]. Multiple lines of evidence indicate that long-lasting up- or downregulation of functional synaptic strengths, referred to as long-term potentiation (LTP) and long-term depression (LTD), respectively, are fundamental synaptic mechanisms underlying hippocampal contributions to these processes. Thus, dendritic and synaptic abnormalities in the hippocampus, either morphological or functional, would be expected to significantly impact spatial cognition. Indeed, neuropsychological investigations requiring the use of spatial information in problem solving indicate that deficits in hippocampal-mediated learning and memory processes are hallmarks of DS [17, 18]. In this paper, we will provide an overview of the morphological and behavioral evidence for altered synaptic plasticity in DS with a focus on the hippocampus and discuss the insights provided by mouse models of this neurodevelopmental disorder into the potential molecular mechanisms contributing to these deficits.

## 2. Evidence for Altered Synaptic Plasticity in DS: A Neurodevelopmental Impact

The basis for altered synaptic plasticity in DS can be found in changes in the physical structure of the dendrites. Alterations in the shape and densities of dendrites would be expected to adversely affect the information storage capacity of neural networks by reducing the number of potential sites for plasticity to occur. Consistent with this idea and the observed deficits in cognition associated with DS, examination of postmortem brain tissue from DS individuals reveals profound alterations in dendritic and neuronal densities and morphology across many regions of the brain beginning *in utero* and persisting throughout life. The neocortical development of DS fetuses appears normal up to at least gestational week 22 [19–21]. By 40 weeks gestation, less discrete lamination is observed in the neocortex of DS fetuses with lower and higher cell densities observed in the visual cortex and superior temporal neocortex, respectively [19, 20]. In the hippocampus, deficits begin to appear slightly earlier as DS fetuses (17 to 21 weeks of gestation) show altered morphology, reduced neuron numbers, enhanced apoptosis, and reduced cell proliferation [22–24]. These changes may result, in part, from reductions in serotonin, dopamine, and GABA levels in the fetal DS cortex [25] since, during development, neurotransmitters such as these can act as neurotrophic factors assisting with neuronal migration, axon guidance, and neurite development [26]. 

 During the early postnatal period, significant deficits in brain weight and gross morphology as well as myelination and neuronal densities and morphology appear [27]. Initially, dendritic expansion is enhanced in DS infants, but, by the first to second year of life, this trend reverses to become a deficit [19, 28] which persists into adulthood [19, 29]. Dendritic spine numbers are reduced, and morphology altered in DS [30, 31]. Consistent with adverse changes in dendrite morphology, synaptogenesis is also aberrant in DS fetuses [19, 32, 33] and remains deficient in adulthood [34]. MRI studies reveal that DS children and young adults have smaller overall brain volumes [35, 36] with particular deficits noted in the hippocampus [36, 37]. Hippocampal volume, that continues to decrease with age in DS individuals [38], was found to be inversely correlated with the degree of cognitive impairment [36]. Cognitive tests such as the Cambridge Neuropsychological Testing Automated Battery (CANTAB) and the Arizona Cognitive Test Battery (ACTB), the latter specifically tailored to address DS deficits, indicate that hippocampal function is particularly impacted by the DS genetic condition [17, 39].

These morphological and cognitive deficits are consistent with aberrant synaptic plasticity, and, indeed, while difficult to measure directly in human subjects, evidence suggests that plasticity is reduced at least in the motor cortex of DS individuals [40]. Additionally, functional MRI (fMRI) during cognitive processing tasks reveals abnormal neural activation patterns in DS children and young adults [41, 42]. Examination of resting glucose metabolism in the cerebral cortex of DS individuals found enhanced uptake in brain regions associated with cognition suggesting cellular hyperactivity in those areas [43]. To better understand the functional consequences resulting from altered network morphologies as well as investigate potential alterations in intracellular signaling cascades contributing to aberrant plasticity, it was necessary to develop and then examine animal models of DS. 

## 3. Modeling DS Cognitive Impairment

Over the past few decades, several mouse models of Down syndrome have been developed to further our understanding of the link between enhanced gene dosage and DS phenotypes such as altered plasticity and cognition. The Tc1 mouse model carries an almost complete, freely segregating copy of Hsa21, but the chromosome is present in only approximately 50% of cells making this a mosaic model of DS [46]. Interestingly, some genes have been deleted from the “inserted” Hsa21 [47]. It is important to note that, in spite of the mosaicism and gene deletions, many DS phenotypes have been replicated in this model [46, 48, 49]. Other mouse models have taken advantage of the homology between regions of Hsa21 and mouse chromosomes 10, 16, and 17 (Mmu10, 16, 17) making models in which these genes are triplicated highly useful in understanding the genetic basis of DS phenotypes [50, 51]. A mouse model trisomic for all Hsa21 homologous segments was recently developed and holds great promise for furthering our understanding of DS [52]. As this is a relatively new model, however, most research has been conducted using the Ts65Dn segmental trisomic mouse [53–55] which is trisomic for more than 50% genes of Hsa21 homologs [56, 57] and has well-documented DS-like deficits in behavioral tasks such as those relying upon declarative memory (novel object recognition and spontaneous alternation tasks) and the proper encoding and recollection of spatial information (radial arm and Morris water mazes) [58–63]. While the Ts65Dn mouse is the only mouse model of DS to have a freely segregating supernumerary chromosome, they are also trisomic for 60 genes that do not have Hsa21 homologs [64], and the impact of overexpression of these genes on Ts65Dn phenotypes remains to be determined. 

Similar to the Ts65Dn mouse but with smaller triplicated Mmu16 segments are the Ts1Cje and Ts1Rhr mouse models. These mice display phenotypes similar to Ts65Dn mice including hippocampal dysfunction; however, the severity of the deficits is reduced [65–69]. The reduced severity of DS-like deficits in mice with fewer trisomic genes highlights one of the powerful aspects of mouse models: the ability to control expression of certain HSA21 homologs to assess their contribution to specific DS phenotypes. Those deficits associated with the hippocampus, whose function is notably altered in DS individuals [17, 39], will be the focus of the remainder of this paper.

### 3.1. Morphological Changes

Mouse models of DS, including the Ts65Dn strain, show similar detrimental changes in neuronal and dendritic morphologies observed in humans. The neocortex of Ts65Dn mice contains fewer excitatory neurons but an increased number of a subset of inhibitory neurons relative to euploid controls, a phenotype that was reversed by normalizing the expression levels of *Olig1/2* [70]. Additionally, regions both in the neocortex and hippocampus have decreased spine densities with larger spine volumes [71]. In the dentate granule cells of the hippocampus, there is a shift of inhibitory synaptic connections away from the dendritic shafts and onto the necks [71]. Such a change would be expected to increase the efficacy of inhibitory synaptic transmission given the significantly reduced volume of the spine neck compared to the shaft. At a finer resolution, symmetric (presumed inhibitory synapses) have greater opposition lengths in Ts65Dn while asymmetric synapses were unaltered [72], again supporting a shift towards excess inhibition in these mice. Similar but less severe changes are observed in Ts1Cje mice [67]. Beyond suppressing excitatory synaptic activity, the altered spine morphology and shift towards excess inhibition in trisomy would be expected to suppress plasticity-related signaling cascades that frequently rely on depolarization-mediated calcium influx into the postsynaptic structural domains.

### 3.2. Functional Changes

**Figure 1:**
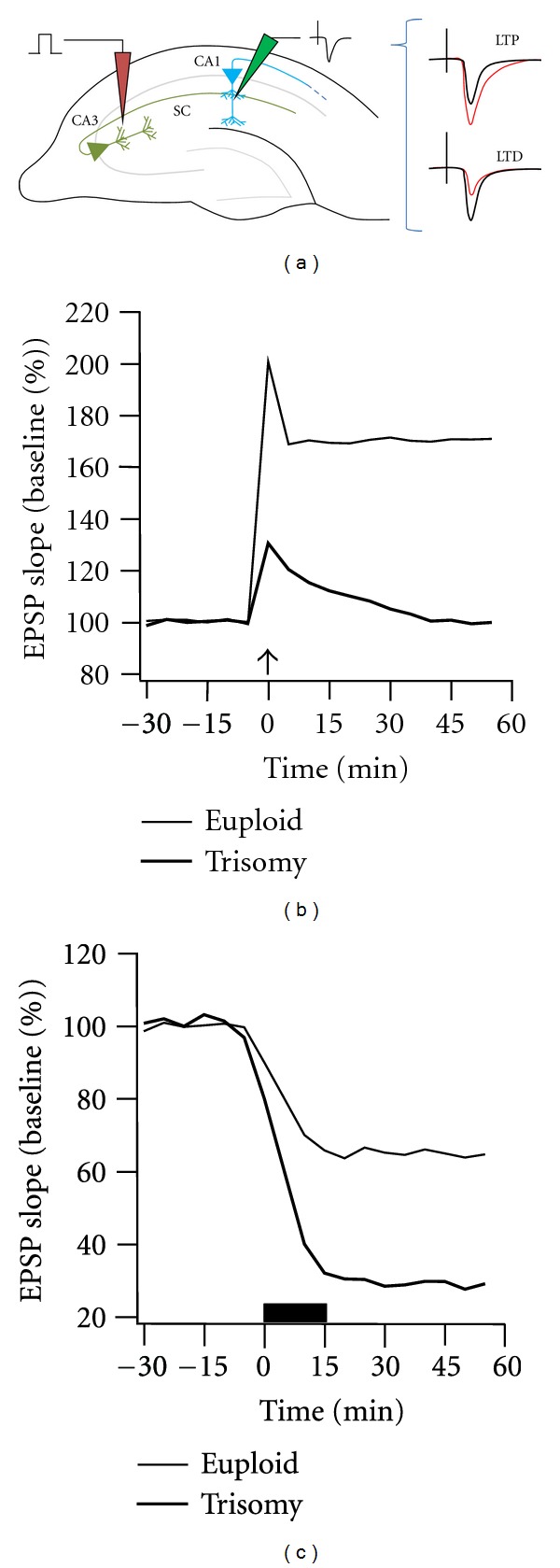
Depiction of altered CA1 hippocampal plasticity in Ts65Dn mice. (a) Diagram indicating electrode placement for stimulating Schaffer collaterals arising from CA3 and recording the evoked field excitatory postsynaptic potential (EPSP) in CA1. Traces to the right indicate the typical change in evoked responses (red) following LTP and LTD. (b) Simulated data depicting suppressed LTP in Ts65Dn mice. After high-frequency stimulation of SC (at arrow head), the field EPSP increases and remains enhanced in euploid mice but fails to remain elevated in Ts65Dn mice. (c) Simulated LTD data depicting exaggerated depression of evoked EPSPs following low-frequency stimulation of SC in Ts65Dn mice. (Traces in B and C based on data from [44, 45].)

Synaptic plasticity in the hippocampus is often investigated in the context of long-term potentiation (LTP) in which high-frequency activation of specific inputs in the hippocampus results in a long-lasting potentiation of synaptic responses along the excited afferent pathway. First described in the anesthetized rabbit [73], this phenomenon is believed to be a fundamental mechanism underlying memory formation [8, 74] and is suppressed in Ts65Dn (depicted in Figure 1 for the CA1 region of the hippocampus) [44, 75, 76] and Ts1Cje [66, 67] but not in Ts1Rhr mice [69] (however, see [68]) as well as mice trisomic for Hsa21 syntenic regions of Mmu16 and Mmu17 [77] or those carrying an almost complete copy of Hsa21 [46, 48]. 

As outlined above, structural changes suggest that inhibition is exaggerated in the trisomic hippocampus. Consistent with this idea is the observation that LTP in the dentate gyrus and CA1 regions of Ts65Dn hippocampal slices, induced by high-frequency stimulation and theta burst protocols, respectively, can be rescued by the GABA_A_ antagonists picrotoxin [75, 76]. Blockade of GABA_A_ receptors in hippocampal slices from Ts1Cje and Ts1Rhr mice rescues LTP deficits in the dentate gyrus in these DS mouse models as well [67, 68]. A similar treatment in Ts65Dn mice leads to an enhancement in cognitive performance [60].

In addition to suppressed LTP, hippocampi from Ts65Dn mice show enhanced long-term depression (LTD) in response to sustained activation of excitatory synapses [45, 78]. This latter effect can be reversed with the uncompetitive NMDA receptor antagonist memantine [78] and also improves the cognitive performance of Ts65Dn mice [79–81]. These results draw a clear link between altered synaptic plasticity in the hippocampus and cognitive performance in the Ts65Dn mouse model of Down syndrome. 

### 3.3. Synaptic-Plasticity-Related Signaling Cascades

**Figure 2:**
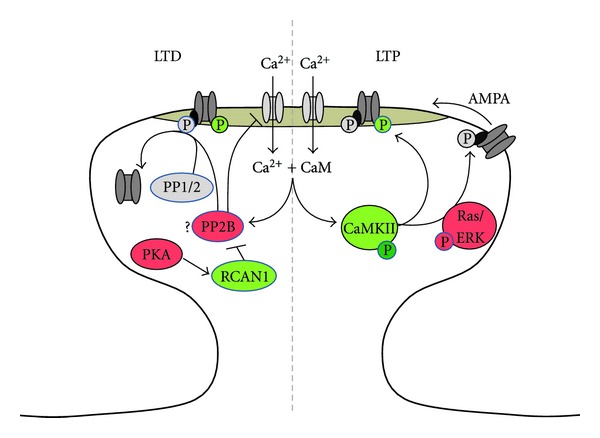
Alterations in intracellular signaling cascades affecting postsynaptic AMPAR response in Ts65Dn hippocampus. Green indicates elevated levels/activity at baseline, while red indicated diminished activity. During LTP (right), enhanced CaMKII and GluR1 subunit phosphorylation in Ts65Dn synapses may result in a saturated condition incapable of additional potentiation. Reduced ERK activity may reduce migration of new AMPARs into the PSD. In LTD, overexpression of RCAN1 should reduce the activity of PP2B (calcineurin) resulting in reduced internalization of AMPA receptors and potential reduction of NMDAR mean open times. Rescue of LTD in Ts65Dn mice by NMDAR antagonists suggests enhanced NMDAR activity contributes to altered LTD through yet unidentified mechanisms.

Changes in intracellular calcium concentrations are important triggers for many intracellular signaling cascades including those underlying LTP and LTD [82]. For example, the presence of a calcium chelator that buffers intracellular calcium levels in postsynaptic neurons prevents the induction of LTP [83] consistent with the hypothesis that a postsynaptic rise in intracellular calcium levels is necessary for LTP [8]. When strongly depolarized, the magnesium block of NMDA channels is lifted providing the main (but not exclusive) mechanism for calcium entry into the postsynaptic cell. Elevated intracellular calcium levels trigger a cascade of intracellular messengers that ultimately lead to the induction and maintenance of synaptic plasticity (both LTP and LTD depending on the kinetics). An excellent overview of this process can be found in several reviews [82, 84], and only key components known to be affected by trisomy (Figure 2) will be discussed here.

#### 3.3.1. CaMKII

Activation of postsynaptic NMDA receptors (NMDARs) concomitant with the depolarization of the postsynaptic membrane is sufficient to relieve the magnesium block of NMDA channels leading to an influx of calcium into the intracellular postsynaptic space. In the case of LTP, the rise of intracellular calcium leads to the activation of calcium calmodulin-dependent protein kinase II (CaMKII), a necessary step for initiating NMDAR-dependent LTP [82]. Blocking CaMKII prevents induction of LTP, [82, 85, 86], while constitutively active forms can induce LTP [87]. During all phases of LTP (induction, early, and late), levels of phosphorylated CaMKII are increased in the hippocampus [88]. CaMKII phosphorylated at threonine 286 (Thr286) can become constitutively active providing a potential switch for initiating and then maintaining potentiation [89]. Alternatively, phosphorylation of Thr305/306 can inhibit the expression of LTP by interfering with the binding of calcium/calmodulin [90, 91]. Indeed, cognitive deficits associated with Angelman syndrome were reversed in a mouse model of the disorder by reducing the levels of CaMKII phosphorylated at Thr305/306 [92]. Thus, depending on the site of phosphorylation, CaMKII can facilitate or suppress initiation and maintenance of LTP. In Ts65Dn mice, we found that baseline levels of CaMKII phosphorylated at Thr286 are elevated in the hippocampus [93]. Excessive basal phosphorylation of the CaMKII site leading to constitutive activation could leave the DS modeling trisomic network in a saturated state unable to shift to more potentiated levels.

One of the substrates targeted by CaMKII during the initial expression of LTP is the serine 831 residue on GluR1 subunits of AMPA receptors [94, 95]. This phosphorylation leads to an increase in conductance of the AMPA channel [96] providing a rapid mechanism for enhancing glutamatergic synaptic strength. In Ts65Dn mice, we find that baseline levels of phosphorylated serine 831 in synaptically located GluR1 receptors are elevated [93]. This apparent increase in AMPA channel conductance appears not to have any significant effect on baseline excitatory synaptic transmission which is normal in the Ts65Dn hippocampus [45, 75, 76, 93]. However, it could also partially occlude the initiation of LTP in these mice by leaving Ts65Dn hippocampal excitatory synapses with fewer AMPA channels available for potentiation. This finding would be consistent with our observation of increased CaMKII in Ts65Dn hippocampus noted above [93] and the suggestion that some components of the LTP network are in an apparent saturated state in these mice.

### 3.4. PKA, RCAN1, Calcineurin

Protein kinase A (PKA) also plays a critical role in establishing LTP. In particular, evidence suggests that it is involved in initiating the protein synthesis required for the late phase of LTP [97, 98]. Blocking PKA activity suppresses the late phase of LTP (lasting beyond 3 hours) while leaving the early phase of LTP (less than 3 hours) unaffected [99, 100]. Transgenic mice in which PKA activity is reduced have significantly decreased late-phase LTP in CA1 but normal early LTP and perform poorly on tasks requiring long- but not short-term memory formation [101]. 

PKA plays a role in LTD where its substrates, such as GluR1, show increased dephosphorylation following induction [102, 103]. Dephosphorylation of GluR1 subunits should reduce the conductance levels of affected AMPA receptors [96] resulting in a reduction of synaptic strength. PKA also enhances the activity of RCAN1 [104], an inhibitor of calcineurin which contributes to AMPA receptor internalization [105] and reductions in NMDA receptor mean open time [106]. In Ts65Dn mice, we found that PKA activity is reduced in the hippocampus [93], which should adversely affect LTP by reducing protein expression required for the late phase. With respect to LTD, reduced PKA activity would result in more AMPA receptors remaining in a high conductance state and less facilitation of RCAN1 activity. This latter effect is offset, however, by the overexpression of the gene encoding RCAN1 in DS and Ts65Dn mice [4]. How these factors contribute to the enhancement of LTD in Ts65Dn hippocampi [45, 78] remains to be determined. 

### 3.5. Extracellular Receptor Kinase (ERK)

In addition to GluR1 subunits, both CaMKII and PKA converge on another common effector, the mitogen-activated protein kinase (MAPK/ERK), that is associated with a host of synaptic-plasticity-related cellular processes [107]. In the case of hippocampal LTP, there is a rapid increase in the amount of phosphorylated ERK following induction [108] and blocking ERK activation prevents expression of LTP [109]. Cultured hippocampal neurons undergo phosphorylated ERK-dependent spine generation following LTP conditioning stimuli implicating this pathway in spine formation [110]. Additionally, it is believed that lateral diffusion of extrasynaptic AMPA receptors containing GluR1 subunits into the postsynaptic density (PSD) is a major contributor to LTP expression [111]. This process is assisted by Ras/Erk phosphorylation of stargazin on extrasynaptic AMPA receptors enabling them to be structurally secured at the synapse to PSD95 [112]. In the Ts65Dn hippocampus, ERK phosphorylation is decreased [93] suggesting decreased activity. This would be expected to adversely affect the insertion of new AMPA receptors into the PSD as well as morphological restructuring of synaptic spines observed after LTP in normal mice [113, 114]. 

### 3.6. BDNF Pathway

Brain-derived neurotrophic factor (BDNF) contributes to LTP by stimulating protein synthesis. In activating postsynaptic TrkB receptors, BDNF stimulates the PI3K pathway [115] which can initiate translation through mammalian target of rapamycin (mTOR) thereby enhancing synthesis of proteins such as CaMKII*α*, GluR1, and NMDA receptor subunit 1 [116]. In Ts65Dn mice, we found that PI3K phosphorylation failed to increase following an LTP induction protocol suggesting this pathway is perturbed by trisomy [93]. Consistent with this notion, in DS individuals, BDNF blood plasma levels are approximately 5 times higher than in age-matched controls [117]. As BDNF readily crosses the blood-brain barrier [118], these levels likely reflect those present in the CNS as well. 

Examination of BDNF levels in DS mouse models presents a complex picture. In Ts65Dn mice, levels of BDNF in the frontal cortex are diminished [119]. In the hippocampus, both no difference [81] and a reduction [120] compared to control have been reported. In the latter case, the reduction in BDNF levels was associated with decreased neurogenesis and was reversible through treatment with fluoxetine [120]. In the Ts1Cje mouse model of DS, BDNF is overexpressed in the hippocampus, particularly in the dentate gyrus and CA1 regions and in the dendrites of dissociated hippocampal neurons grown in culture [121]. Increased BDNF levels in Ts1Cje mice hippocampi were associated with greater levels of phosphorylated Akt-mTOR and expression of GluR1 protein which could not be further enhanced with exogenous supplemental BDNF suggesting this pathway related to synaptic plasticity is saturated in these mice preventing further contributions to LTP [121]. 

The discrepancies between observations in Ts65Dn and Ts1Cje BDNF levels may reflect how BDNF expression is distributed in these structures, elevated in some subregions or subcellular compartments while diminished in others, resulting in an increased functional effect despite reduced global levels. Conversely, the differences in observed BDNF levels could be related to the different numbers of genes overexpressed in these two mouse lines [53, 65, 122] or, as mice of differing age groups were used in the studies, may reflect differences in expression levels as a function of age. Further investigation is necessary to fully align these observations. However, the observation that rapamycin has a restorative effect on phosphorylated Akt-mTOR levels in Ts1Cje suggests a potential therapeutic mechanism for improving cognition in DS individuals [121] possibly by normalizing a pathway involved in synaptic plasticity. 

### 3.7. GABA_B_-GIRK2 Attenuation of Synaptic Plasticity

As mentioned above, postsynaptic calcium influx is critical for LTP and LTD in the hippocampus. This initiating step relies heavily upon depolarization of the postsynaptic membrane to relieve the voltage-dependent magnesium block of NMDA channels. Any phenomenon that reduces the ability of the postsynaptic membrane to depolarize would thus be expected to adversely affect plasticity. Through its coupling to *GABA *_B_ receptors, the type 2 G-protein-activated inward rectifying potassium (GIRK2) channel may act to dampen the expression of LTP in Ts65Dn hippocampus through a shunting mechanism. 

GIRK2 is encoded by the gene *Kcnj6* which is located on the chromosomal segment triplicated in DS and Ts65Dn mice, and, consequently, elevated expression levels have been found in the Ts65Dn hippocampus [50, 123]. At a cellular level, overexpression of GIRK2 leads to a more hyperpolarized resting potential in cultured hippocampal neurons [124] and CA1 pyramidal neurons *in vitro *[125]. Selectively reducing the expression level of GIRK2 by crossing euploid and Ts65Dn mice with mice heterozygous for GIRK2 (GIRK2^+/−^) resulted in a gene dosage-dependent change in the resting membrane potential and facilitation of LTP in GIRK2 knockout mice [50]. Selective overexpression of GIRK2 alone in mice results in cognitive deficits, reduced depotentiation (a functional reversal of potentiation at a synapse), and enhanced LTD [126]. 

These effects on LTP and LTD could be mediated through GABA_B_ receptors which, in pyramidal neurons, are in closest proximity to GIRK2-contaning potassium channels near glutamatergic synapses on dendritic spines [127]. GABA_B_ receptors are functionally linked to GIRK channels, and, indeed, whole-cell GABA_B_-mediated potassium currents are exaggerated in Ts65Dn hippocampal neurons [50, 124, 128]. In CA1, these exaggerated currents have a greater functional impact on the distal dendrites of pyramidal neurons as opposed to those located more proximally [128]. A similar enhancement of GABA_B_-mediated currents is also found in the dentate gyrus where the presynaptic release probability of GABA is increased [129]. Thus, GIRK channels, activated by GABA_B_ and other G-protein coupled receptors, appear to act as a break on synaptic plasticity in the Ts65Dn hippocampus.

## 4. Potential Impact of Altered Plasticity on Hippocampal Processing

**Figure 3:**
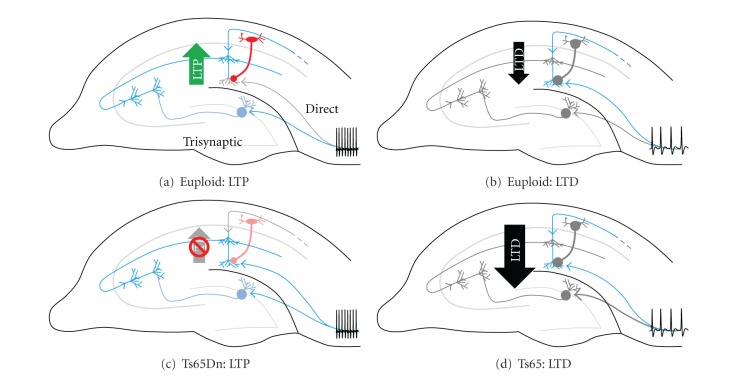
Potential impact of altered synaptic plasticity on hippocampal processing in Ts65Dn mouse model of Down syndrome. Schematic of two main pathways through hippocampus arriving from the entorhinal cortex: temporoammonic (TA)—direct to CA1 distal dendrites; trisynaptic pathway from DG through CA3 to proximal CA1 dendrites. LTP and LTD are proposed to minimize interference between the two pathways [50, 131]. (a) In euploid hippocampi, high-frequency inputs induce LTP in CA1 resulting in enhanced suppression of inputs from TA by feed-forward inhibition arising from interneurons in stratum oriens. (b) Low-frequency inputs depress the trisynaptic pathway releasing distal CA1 dendrites from feed-forward inhibition and allowing information to flow through the TA pathway. (c) In Ts65Dn hippocampi, aberrant LTP in CA1 results in diminished feed-forward inhibition during high-frequency activity allowing TA inputs to become superimposed on those flowing through the trisynaptic pathway. (d) Enhanced LTD would be expected to facilitate flow of low-frequency information through the direct TA pathway in Ts65Dn mice.

The hippocampus receives major inputs from the entorhinal cortex (EC) which converge on CA1 pyramidal through two main pathways: the perforant pathway (PP) and the temporoammonic (TA) pathway [130]. The PP pathway passes through the dentate gyrus to pyramidal neurons in CA3 before impinging upon the relatively proximal dendrites of CA1 pyramidal neurons in stratum radiatum (SR). Conversely, inputs to CA1 from TA target the distal dendrites located in stratum lacunosum molecular (SLM). In the normal hippocampus, frequency-based synaptic plasticity at the CA3-CA1 synapse, coupled with a feed-forward inhibition loop from stratum oriens alveus interneurons that suppress inputs to distal CA1 dendrites, enables segregation of information flow through these two pathways [131]. During high-frequency synaptic activity, the CA3-CA1 synapse would be expected to undergo LTP, increasing the excitatory drive to CA1 pyramidal neurons and, consequently, enhanced suppression of inputs to distal CA1 dendrites by the feed-forward inhibition loop (Figure 3(a)). Thus, TA inputs that target distal CA1 dendrites would be suppressed, and information flows through the CA3-CA1 pathway enhanced during during high-frequency events. Conversely, during low-frequency synaptic activity, the CA3-CA1 synapse would be expected to undergo LTD and become less effective. Inhibition of distal CA1 synapses would then be decreased and information flow through the TA pathway would likely be enhanced (Figure 3(b)). Diminished LTP resulting from trisomy would then interfere with this frequency-based segregation of information flow through the hippocampus. Without LTP, feed-forward inhibition would cause suppression of information flow through the TA pathway causing inputs from the two pathways to become superimposed upon and interfere with each other (Figure 3(c)). In contrast, the flow of information during low frequency signaling would likely remain intact, or potentially facilitated, since enhanced LTD at CA3-CA1 would prevent interference from this pathway (Figure 3(d)).

Electroencephalogram (EEG) recordings from DS individuals suggest that such a preferential suppression of high-frequency information flow may result from overexpression of Hsa21 genes. Compared to controls, DS individuals have increased power at low EEG frequencies and a corresponding reduction in power at higher frequencies [132]. Similar observations have been reported in Ts65Dn mice [133]. While it is not clear that hippocampal activity is accurately reflected in EEG recordings, abnormal EEGs findings are consistent with aberrant processing of high-frequency information in DS individuals and Ts65Dn mice.

## 5. Cognitive Therapies Targeting Plasticity

A number of studies using the Ts65Dn mouse model of DS have examined the possibility of pharmacologically reversing cognitive deficits (reviewed in [122]). Of particular note with respect to hippocampal plasticity are those targeting the excess GABAergic inhibitory tone or NMDA receptors whose activation, as outlined above, is a critical step in initiating LTP and LTD. 

Application of the GABA_A_ receptor antagonist, picrotoxin to hippocampal slices from Ts65Dn mice rescues LTP in the dentate gyrus [75] and CA1 region [76]. Chronic administration of low doses of picrotoxin or other GABA_A_ receptor antagonists (pentylenetetrazole or bilobalide) improves cognition in Ts65Dn mice suggesting that the efficacy of this class of pharmacological agents could be tested for reversing impaired cognition in DS [60]. However, as overinhibition of GABA_A_ receptors can induce seizures, translating these findings to humans requires great caution. Careful screening of similar drugs or design of pharmacological compounds with similar blocking capabilities but reduced propensities for inducing seizures may prove to be effective treatments. Currently, a small molecule targeting GABA_A_ receptors developed by F. Hoffmann-La Roche Ltd (Pharmaceutical pipeline molecule RG1662 http://www.roche.com/roche_pharma_pipeline.htm) is in clinical trials with the goal of safely improving cognition in DS individuals. 

Braudeau et al. [134, 135] are currently investigating a similar promising inhibitor that targets the alpha-5 subunit of GABA_A_ receptors.

Another pharmacological avenue targets aberrant NMDA receptor-mediated signaling apparently present in Ts65Dn mice. The uncompetitive NMDA receptor antagonist memantine improves the cognitive performance of Ts65Dn mice [79] and normalizes hippocampal LTD [78]. Memantine is an FDA approved and fairly well-tolerated drug already in use for treating dementia in Alzheimer disease. Clinical trials assessing the safety, tolerability, and efficacy in alleviating DS cognitive phenotypes are currently underway [57]. 

In addition to pharmacological approaches, behavioral therapies have been shown to improve cognition in Ts65Dn mice. When housed in enriched environments (larger cage with novel objects such as toys and running wheels), trisomic mice performed as well as euploid littermates in the Morris water maze and had normalized hippocampal LTP [136, 137]. Interestingly, environmental enrichment was effective for trisomic females but not trisomic males potentially due to social and physical factors associated with the new environments [138]. 

The benefits of environmental enrichment appear to be linked in part to regulation of excess inhibition in the neocortex and hippocampus. Release of GABA from synaptosomes isolated from the hippocampus and neocortex is elevated in Ts65Dn mice, an effect that is reversed by environmental enrichment [137]. In adult rats with amblyopia (via monocular deprivation during critical period), environmental enrichment reversed visual deficits reduced GABA levels in the visual cortex contralateral to the deprived eye while increasing plasticity [137]. It thus appears possible to regulate aberrant levels of inhibition in trisomic mice behaviorally without pharmacological intervention and achieve similar behavioral outcomes without the concerns associated with nonspecific actions or adverse side-effects of drugs. 

Deficits in neurogenesis in the dentate gyrus and forebrain subventricular zone in Ts65Dn mice are also reversed following environmental enrichment [139] and may add an additional therapeutic layer to the beneficial effect of a decrease in inhibitory tone. A structural benefit of environmental enrichment appears lacking; however, as, unlike euploid mice, this treatment has failed to significantly increase dendritic branching and spine density in Ts65Dn mice [140].

Early behavioral intervention techniques designed to improve development in DS children show great promise [141, 142] suggesting that this comparatively easily translatable therapeutic approach, either used alone or in combination with pharmacological agents, could potentially increase cognitive capacities in DS individuals.

## 6. Conclusion

Synaptic plasticity is believed to be the process central to learning and memory. This belief is bolstered by experiments where drugs that normalize aberrant plasticity in hippocampal slices isolated from mouse models of DS also confer improvements in cognition in in intact adult mice. The initiation and maintenance of plastic changes involve structural and compositional modifications of synapses that depend on intracellular signaling cascades. The reduced excitatory neuronal densities and deficits in dendritic morphologies present in individuals with DS diminish the capacity of their neural networks in general to undergo neuroplastic adaptations. Combined with the deficits in signaling pathways reported in Ts65Dn mice, evidence strongly suggests that synaptic plasticity is severely impaired in DS neural networks. By understanding how plasticity is perturbed, we can design therapies to reverse these phenotypes and ultimately improve cognition in DS individuals.
